# Obituary

**Published:** 1893-03

**Authors:** J. Taft, N. S. Hoff


### Obituary.

Died in Xenia, O., Wednesday, February 15th, Dr. George Watt, aged seventy-two years and eleven months.

Dr. Watt, as is well known to the profession, has been an invalid for many years, and for the past three years has been very feeble. During the war he was assistant surgeon in the army, and while there received injuries from which he never wholly recovered. The result of these was a partial paralysis, which remained and increased upon him during his life. During the last three or four years there has been a rapid breaking down of the entire system, and to this general giving way is due his death.

Dr. Watt was widely known throughout the profession, and though he has not been in a condition for several years to render service to the profession, yet his death will produce sadness in the hearts of all those who have ever known him.

The next number of the REGISTER will contain a more extended notice of the life of Dr. Watt.

The following from the Faculty of the College of Dental Surgery of the University of Michigan has just come to hand :

"In the death of Dr. George Watt, of Xenia, this Faculty feels that in addition to the personal sense of loss of each member, that the dental profession of the world is called upon to mourn his departure ; and that teachers and educational institutions everywhere acknowledge their indebtedness to him for his devotion to the scientific as well as the technical departments of our profession, and his love and work for the dental profession has endeared him to all its members, and they will always remember him with a peculiar appreciation. A good and great man has gone from among us.

"It is with profound sorrow that we learn of his death, and we most cordially extend to his bereaved family our sincere sympathy in their great sorrow."

Signed, "J. TAFT, *Dean.*
"N. S. HOFF, *Sec'y.*"

"February 16th, 1893.

